# Dysembryoplastic neuroepithelial tumors: A model for examining the effects of pathology versus seizures on cognitive dysfunction in epilepsy

**DOI:** 10.1111/epi.12425

**Published:** 2013-10-28

**Authors:** Sallie Baxendale, Elizabeth Donnachie, Pamela Thompson, Josemir W Sander

**Affiliations:** * Department of Clinical and Experimental Epilepsy, UCL Institute of Neurology, NIHR University College London Hospitals Biomedical Research CentreLondon, United Kingdom; † Epilepsy SocietyChalfont St Peter, Buckinghamshire, United Kingdom; ‡ Epilepsy Institute in The Netherlands Foundation (SEIN)Heemstede, The Netherlands

**Keywords:** Dysembryoplastic neuroepithelial tumor, Memory, IQ, Cognitive function, Seizures

## Abstract

**Purpose:**

Dysembryoplastic neuroepithelial tumors (DNTs) provide a unique model for studying the effects of seizures on cognitive development. Epilepsy and antiepileptic medications are prominent features in the lives and schooling of people who develop seizures in childhood. People with an adult onset share the same underlying brain pathology, but their childhood development is unaffected by seizures. Therefore, DNTs provide a model to examine the specific influence of seizures and their treatment on cognitive development, over and above the effects of the underlying pathology in epilepsy.

**Methods:**

We examined the neuropsychological characteristics of 56 adults with DNT and medically intractable epilepsy (mean age 32.7 years). Twenty-two adults (39%) had an age of onset of epilepsy before the age of 12 years (childhood-onset group). Scores on tests of intelligence (Verbal IQ and Performance IQ), reading, working memory, verbal learning, verbal recall, visual learning, and expressive and receptive language ability were analyzed.

**Key Findings:**

There were no significant localization effects (right vs. left vs. extratemporal) on any of the neuropsychological test scores. In the group as a whole, the neuropsychological test scores were significantly lower than healthy, age-matched controls on measures of Verbal IQ (p < 0.01), naming p < 0.01, verbal learning (p < 0.01), and working memory (p < 0.05). The childhood-onset group had significantly lower scores on the measures of Verbal IQ (p < 0.01), Performance IQ (p < 0.05), reading (p < 0.05), naming (p = 0.05), and verbal retention (p < 0.05) than those with an onset of seizures at the age of 12 or older.

**Significance:**

The traditional pattern of lateralized memory deficits seen in people with hippocampal sclerosis may not be present in people with temporal lobe epilepsy associated with a DNT. The presence of seizures and their treatment in early childhood may adversely influence the development of these core cognitive abilities, resulting in patterns of cognitive deficits that remain apparent in adulthood.

First described by Daumas-Duport et al. (1988), dysembryoplastic neuroepithelial tumors (DNTs) are cortically based, hypodense lesions with no mass effect or associated edema. DNTs are associated with medically intractable epilepsy, usually presenting with focal seizures (Daumas-Duport et al., 1988), although generalized seizures have also been reported (Sharma et al., 2009). They are most commonly located in the temporal lobes (Daumas-Duport et al., 1988; Sharma et al., 2009). Complete surgical resection of a DNT is associated with a good prognosis, and higher proportions of Engel class I outcomes have been reported in people who had temporal lobe resections for a DNT than in those with other pathologies (Sharma et al., 2009; Rydenhag et al., 2013).

DNTs were originally thought to be associated with an onset of seizures before the age of 20 (Daumas-Duport, 1993). However, subsequent studies suggest adult-onset seizures in some people. One study (Chang et al., 2010) found that 24% of their series of 50 patients with DNTs had adult-onset seizures. Another study reported a series of 10 patients who had an onset of seizures after the age of 20, with three cases having a late adult onset (35–44 years; Chassoux et al., 2013). Similarly a study of 48 people with DNTs reported that about one third of their sample had an onset of seizures after the age of 20, with 15% after the age of 30. The oldest person in the study was 47 years old at the onset of his seizures (Campos et al., 2009).

Although adult and late adult onset of seizures in DNT are now recognized, the majority of DNT studies have focused on the pediatric population. The neuropsychological characteristics of children with DNTs have been reported in a handful of studies, but few have gone beyond basic intelligence quotient (IQ) measures when examining neuropsychological functioning (Fay-McClymont et al., 2012). Other than IQs in children, the neuropsychological characteristics associated with DNTs have received relatively little attention. Detailed cognitive profiles of adults with DNT have not been reported previously (Raymond et al., 1994; Burneo et al., 2008).

DNTs provide a unique model to study the effects of early versus late onset of seizures in a group with a single pathology that is developmental in nature. Because the tumor is present prior to birth and is frequently found in the temporal lobes, the underlying pathology in both adult- and childhood-onset cases is relatively homogeneous, notwithstanding DNT subtypes (Chassoux et al., 2012). In people with an onset of seizures in childhood, epilepsy and antiepileptic medications are a prominent feature in their development and schooling. People with an adult onset share the same underlying pathology, but their first two decades are unaffected by seizures. Therefore, DNT provides a model to examine the specific influence of seizures and their treatment on cognitive development, over and above the effects of the underlying pathology.

We aimed to explore the impact of a childhood onset of seizures on the neuropsychological profile of adults with DNT. We hypothesized that if an individual completed their core foundation education prior to the onset of seizures, their cognitive function would be more developed in comparison to those with a childhood onset of epilepsy.

## Methods

### Participants

The participants were selected from a database of >1,500 people with epilepsy who have undergone a presurgical evaluation for epilepsy surgery at our center since 1992 and who had completed a neuropsychological assessment. All participants had medically intractable epilepsy and had been referred for surgical evaluation. DNTs were identified on the magnetic resonance imaging (MRI) scans (Raymond et al., 1994) of 26 female and 30 male (n = 56) patients. At the time of the analyses, 33 of the patients had proceeded to surgery with histopathologic confirmation of DNT in the resected tissue, where available. The group had a mean age of 32.7 years (standard deviation [SD] 10.9). The demographic and clinical characteristics of the sample are presented in Table 1.

**Table 1:** Demographic and clinical characteristics of the sample

Pathology	Right temporal lobe (RTL) – n = 28
	Left temporal lobe (LTL) – n = 20
	Extratemporal (ExT) – n = 8 (frontal n = 6, parietal n = 1, occipital n = 1)
Age of onset	Before 12 years (n = 22)
	Age 12–20 years (n = 19)
	Age 21–30 years (n = 9)
	Age >31 years (n = 6)

### Controls

A control group of 604 patients with unilateral hippocampal sclerosis identified on MRI were selected from the same database (n = 281 right hippocampal sclerosis [RHS]; 323 left hippocampal sclerosis [LHS]). The hippocampal sclerosis [HS] group did not differ significantly from the DNT group in age and had a mean age of 34.4 years (SD 9.9).

### Neuropsychology

All participants underwent a neuropsychological assessment as part of their presurgical assessment. Scores from the following tests were used as a measure of function in each cognitive domain.

1. Intellectual function (Verbal IQ [VIQ] and Performance IQ [PIQ] from the Wechsler Adult Intelligence Scales)
2. Reading (the National Adult Reading Test)
3. Working Memory (Digit Span from the Wechsler Scales)
4. Naming (Graded Naming Test; McKenna & Warrington, 1980)
5. Verbal Comprehension (De Renzi Token Test)
6. Verbal Learning (List Learning from the Adult Memory and Information Processing Battery, AMIPB; Coughlan & Hollows, 1985)
7. Verbal Recall (Delayed Story Recall from the AMIPB)
8. Visual Learning (Design learning from the AMIPB)

The scores on these tests were converted to *z*-scores based on the published norms for each test to allow comparison between tests and with aged-matched norms from healthy controls.

### Analyses

Localization effects (right temporal lobe [RTL] vs. left temporal lobe [LTL] vs. extratemporal [ExT]) on the neuropsychological scores were explored with a multivariate analysis of variance (MANOVA). The DNT group norms were compared to the published healthy control norms using one-sample *t*-tests.

The participants were divided into two groups. Those with an age of onset prior to the age of 12 (early onset; n = 22) and those with an onset of seizures at the age of 12 or older (later onset; n = 34). The age of 12 was used as a cutoff, as this represents the age at which primary education ends in the United Kingdom. We hypothesized that children who had completed their primary education without the disruptive influence of seizures on the acquisition of their core basic skills (numeracy, literacy, and problem solving) would have less extensive neuropsychological difficulties in adulthood than those whose early cognitive development occurred in the context of seizures and treatment for epilepsy.

## Results

### Neuropsychological profiles

*z*-scores on the neuropsychological tests for the DNT groups (RTL, LTL, ExT) are presented in Table 2.

**Table 2:** Mean *z*-scores on the neuropsychological test scores by localization (whole group, RTL, LTL, and ExT) and age of onset (before the age of 12 and at the age of 12 or older)

	RTL N = 28	LTL N = 20	ExT N = 8	Whole group N = 56	One sample *t*-test (healthy controls comparison vs. DNET)	Childhood onset N = 22	Later onset N = 34	Early onset vs. later onset
Verbal IQ: mean	−0.22	−0.67	−0.65	−0.44	t = −3.4, p = 0.001	−0.80	−0.21	t = −2.3, p = 0.01
N	27	20	8	55		22	33	
SD	0.81	1.17	0.73	0.96		0.75	1.02	
Performance IQ: mean	0.01	−0.12	−0.18	−0.06	t = −0.4, n.s.	−0.41	0.17	t = −1.9, p < 0.05
N	26	19	7	52		21	31	
SD	1.04	1.22	0.89	1.07		0.83	1.16	
Reading: mean	−0.04	−0.23	−0.33	−0.16	t = −1.3, n.s.	−0.41	−0.00	t = −1.7, p < 0.05
N	22	17	8	47		18	29	
SD	0.75	0.77	1.09	0.81		0.58	0.90	
Working memory: mean	−0.10	−0.38	−0.33	−0.23	t = −1.7, p < 0.05	−0.41	−0.12	t = −1.0, n.s.
N	28	19	8	55		21	34	
SD	0.89	1.22	0.83	1.0		0.97	1.02	
Naming: mean	−0.75	−1.08	−0.75	−0.86	t = −4.8, p < 0.001	−1.34	−0.58	t = −2.1, p = 0.01
N	25	17	7	49		18	31	
SD	1.42	1.06	1.04	1.24		0.93	1.32	
Comprehension: mean	0.023	−0.70	−0.18	−0.26	t = −1.3, n.s.	−0.36	−0.18	t = −0.47, n.s.
N	21	15	8	44		19	25	
SD	0.60	2.00	0.78	1.30		1.72	0.88	
Verbal learning: mean	−0.39	−0.65	−1.04	−0.57	t = −4.5, p < 0.001	−0.56	−0.57	t = 0.04, n.s.
N	28	19	7	54		20	34	
SD	1.00	0.88	0.44	0.92		0.98	0.90	
Verbal recall: mean	0.13	−0.32	0.02	−0.050	t = −24, n.s.	−0.53	0.28	t = −2.0, p < 0.05
N	25	18	6	49		20	29	
SD	1.18	1.80	1.17	1.43		1.61	1.21	
Visual learning: mean	−0.14	−0.86	−0.11	−0.38	t = −1.5, n.s.	−0.60	−0.24	t = −0.69, n.s.
N	28	18	7	53		21	32	
SD	2.12	1.60	0.58	1.85		1.5	2.05	

n.s., not significant, p > 0.05.

There were no statistically significant differences between the RTLE, LTLE, and ExT groups on any of the neuropsychological measures (MANOVA Hoteling Trace F = 0.71, p > 0.05; see Table 2). In the DNT group as a whole, the scores were significantly lower than in healthy, age-matched controls on measures of Verbal IQ (p < 0.01), naming (p < 0.001), verbal learning (p < 0.01), and working memory (p < 0.05).

### Comparison with hippocampal sclerosis controls

In the HS control group, the MANOVA indicated a significant effect of laterality (MANOVA Hoteling Trace F = 2.5, p < 0.01), with the LHS group performing significantly more poorly than the RHS group on measures of verbal learning (p < 0.01) and naming (p = 0.01).

In patients with right temporal lobe epilepsy, the HS group had significantly lower scores than the DNT group on the measures of Verbal IQ, Performance IQ, Verbal Learning, and Visual learning.

In patients with left temporal lobe epilepsy, the HS group had significantly lower scores than the DNT group on measures of Verbal Learning.

### Seizure onset

The childhood-onset DNT group had significantly lower scores on the measures of Verbal IQ (p < 0.01), Performance IQ (p < 0.05), reading (p < 0.05), naming (p = 0.05), and verbal retention (p < 0.05) than those with an onset of seizures at the age of 12 or older (see Table 2). Patients in the early onset group were significantly younger at the time of testing than patients in the later-onset group. (Early onset, mean age = 28.5 years, SD = 8.6; later onset mean age 35.6 years, SD = 11.5; t = −2.4, p < 0.05).

## Discussion

Many factors combine to shape the nature and extent of neuropsychological difficulties in people with epilepsy, including the underlying pathology and factors associated with seizures and their treatment. It is often difficult to disentangle these factors (Baxendale & Thompson, 2010). DNT provides a unique model to study the effects of early versus later onset of seizures in a group with a homogenous, developmental pathology.

Our findings indicate that the traditional pattern of lateralized memory deficits seen in people with temporal lobe epilepsy and hippocampal sclerosis (Baxendale, 2008) may not be present in people with temporal lobe epilepsy and DNT. We did not find any significant differences between the RTL, LTL, and ExT groups on any of the neuropsychological measures of intellect, language, or memory function in this study. It is unclear whether the absence of lateralized deficits in this group reflects the developmental nature of the pathology, and consequent atypical representation of function or the location of the tumor, which in the majority of cases in our series was extrahippocampal in the RTL and LTL groups. Unfortunately the numbers in this study were not sufficient to allow a detailed analyses of the location of the DNT within the temporal lobes with respect to neuropsychological deficits, given the qualitative nature of the lesion descriptions in the radiologic reports available. This is a shortcoming of the retrospective study design. Nevertheless our results have important implications for the clinical interpretation of lateralizing and localizing significance of the neuropsychological profiles of these patients, particularly in presurgical evaluations, and the prediction of postoperative deterioration. It is also likely to have a significant effect on postoperative change in neuropsychological function, particularly if the resection involves the mesial structures, rather than a lesionectomy. Further work is underway to examine the postoperative changes in cognition in this group.

In our sample, an onset of seizures before the age of 12 was associated with a wider range of neuropsychological deficits, affecting general intellectual ability, reading, naming, and verbal retention than a later onset of seizures. These findings indicate that the presence of seizures and their treatment in early childhood may adversely influence the development of these core cognitive abilities. It is unclear whether this is due to the disruptive effects of seizures and antiepileptic medications on brain development, or the effects of frequent seizures on school attendance and disrupted lessons and education. The pattern of neuropsychological deficit we recorded provides evidence for both of these processes. The development of IQ, particularly Performance IQ, is less influenced by educative factors than other cognitive abilities, whereas naming and reading skills are highly correlated with indices of education (Hiscock, 2007; Nisbett et al., 2012). Our study indicates that a seizure onset at the age of 12 or older in patients with DNT protects, to some degree, the development of both these kinds of cognitive ability. It is not possible to dismiss the potential effects of antiepileptic drugs, absence from school, and seizure frequency on cognitive development on in the early onset group of this study.

Our results suggest that the presence of seizures in childhood leads to greater cognitive deficits than those associated with just the underlying pathology. This is consistent with the conclusions of Helmstaedter and Elger (2009), who found that people with an early onset of seizures fail to develop adequate learning and memory performance during childhood. Our data suggest that it is the presence of seizures and/or treatments for epilepsy in childhood that may be responsible for these developmental failures rather than the underlying brain pathology, at least in people with DNT.

Because this was a retrospective study, we did not have accurate data on childhood seizure frequency in the group. We did not address the effects of seizure frequency in this study, just the age of onset of seizure activity. It is methodologically difficult to quantify seizure frequency over a lifespan, but all of the participants in this study had medically intractable seizures at the time of assessment and DNTs are generally associated with poorly controlled epilepsy following the onset of seizures. In an earlier longitudinal study in adults, we found that frequent focal seizures were associated with declines in memory and executive function, whereas periods of remission had a protective effect (Thompson & Duncan, 2005). Taken together the results of these studies indicate that seizures themselves can have a detrimental impact on both the development of cognitive abilities in childhood and the maintenance of function in adulthood.

As in other series, many of the patients in this sample had a long history of epilepsy prior to their evaluation for surgery. Rydenhag et al. (2013) reported that seizure outcome can be improved in these patients if surgery is considered earlier. Further work is underway to see if these benefits also extend to cognitive outcomes in this group. The advantage of studying patients with DNT is that we know that the underlying pathology has been present since birth. This is not the case in hippocampal sclerosis where longitudinal studies have suggested that the pathology can have a bidirectional relationship with seizures. It is therefore very difficult to separate out the effects of underlying pathology from seizures in patients with HS, particularly in retrospective designs such as this. The study of people with DNTs and early versus later onset seizures would also allow similar exploration of the effects of seizures on other critical aspects of development including psychosocial functioning and susceptibility to psychiatric comorbidities in adulthood.

## References

[b1] Baxendale S (2008). The impact of epilepsy surgery on cognition and behavior. Epilepsy Behav.

[b2] Baxendale S, Thompson P (2010). Beyond localization: the role of traditional neuropsychological tests in an age of imaging. Epilepsia.

[b3] Burneo JG, Tellez-Zenteno J, Steven DA, Niaz N, Hader W, Pillay N, Wiebe S (2008). Adult-onset epilepsy associated with dysembryoplastic neuroepithelial tumors. Seizure.

[b4] Campos AR, Clusmann H, Von LM, Niehusmann P, Becker AJ, Schramm J, Urbach H (2009). Simple and complex dysembryoplastic neuroepithelial tumors (DNT) variants: clinical profile, MRI, and histopathology. Neuroradiology.

[b5] Chang EF, Christie C, Sullivan JE, Garcia PA, Tihan T, Gupta N, Berger MS, Barbaro NM (2010). Seizure control outcomes after resection of dysembryoplastic neuroepithelial tumor in 50 patients. J Neurosurg Pediatr.

[b6] Chassoux F, Rodrigo S, Mellerio C, Landre E, Miquel C, Turak B, Laschet J, Meder JF, Roux FX, Daumas-Duport C, Devaux B (2012). Dysembryoplastic neuroepithelial tumors: an MRI-based scheme for epilepsy surgery. Neurology.

[b7] Chassoux F, Landre E, Mellerio C, Laschet J, Devaux B, Daumas-Duport C (2013). Dysembryoplastic neuroepithelial tumors: epileptogenicity related to histologic subtypes. Clin Neurophysiol.

[b8] Coughlan A, Hollows S (1985). Adult memory and information processing battery.

[b9] Daumas-Duport C (1993). Dysembryoplastic neuroepithelial tumours. Brain Pathol.

[b10] Daumas-Duport C, Scheithauer BW, Chodkiewicz JP, Laws ER, Vedrenne C (1988). Dysembryoplastic neuroepithelial tumor: a surgically curable tumor of young patients with intractable partial seizures. Report of thirty-nine cases. Neurosurgery.

[b11] Fay-McClymont TB, Hrabok M, Sherman EM, Hader WJ, Connolly MB, Akdag S, Mohamed IS, Wiebe S (2012). Systematic review and case series of neuropsychological functioning after epilepsy surgery in children with dysembryoplastic neuroepithelial tumors (DNT). Epilepsy Behav.

[b12] Helmstaedter C, Elger CE (2009). Chronic temporal lobe epilepsy: a neurodevelopmental or progressively dementing disease?. Brain.

[b13] Hiscock M (2007). The Flynn effect and its relevance to neuropsychology. J Clin Exp Neuropsychol.

[b14] McKenna P, Warrington EK (1980). Testing for nominal dysphasia. J Neurol Neurosurg Psychiatry.

[b15] Nisbett RE, Aronson J, Blair C, Dickens W, Flynn J, Halpern DF, Turkheimer E (2012). Intelligence: new findings and theoretical developments. Am Psychol.

[b16] Raymond AA, Halpin SF, Alsanjari N, Cook MJ, Kitchen ND, Fish DR, Stevens JM, Harding BN, Scaravilli F, Kendall B (1994). Dysembryoplastic neuroepithelial tumor. Features in 16 patients. Brain.

[b17] Rydenhag B, Flink R, Malmgren K (2013). Surgical outcomes in patients with epileptogenic tumours and cavernomas in Sweden: good seizure control but late referrals. J Neurol Neurosurg Psychiatry.

[b18] Sharma MC, Jain D, Gupta A, Sarkar C, Suri V, Garg A, Gaikwad SB, Chandra PS (2009). Dysembryoplastic neuroepithelial tumor: a clinicopathological study of 32 cases. Neurosurg Rev.

[b19] Thompson PJ, Duncan JS (2005). Cognitive decline in severe intractable epilepsy. Epilepsia.

