# Supplementary material for: Dysembryoplastic neuroepithelial tumors: A model for examining the effects of pathology versus seizures on cognitive dysfunction in epilepsy
Source: Epilepsia. 2013 Oct 28;54(12):2214–8. doi: 10.1111/epi.12425 (PMC4209113; doi:10.1111/epi.12425)
Supplement: Supplementary file 1 [file epi0054-2214-sd1.pptx]

## Slide 1
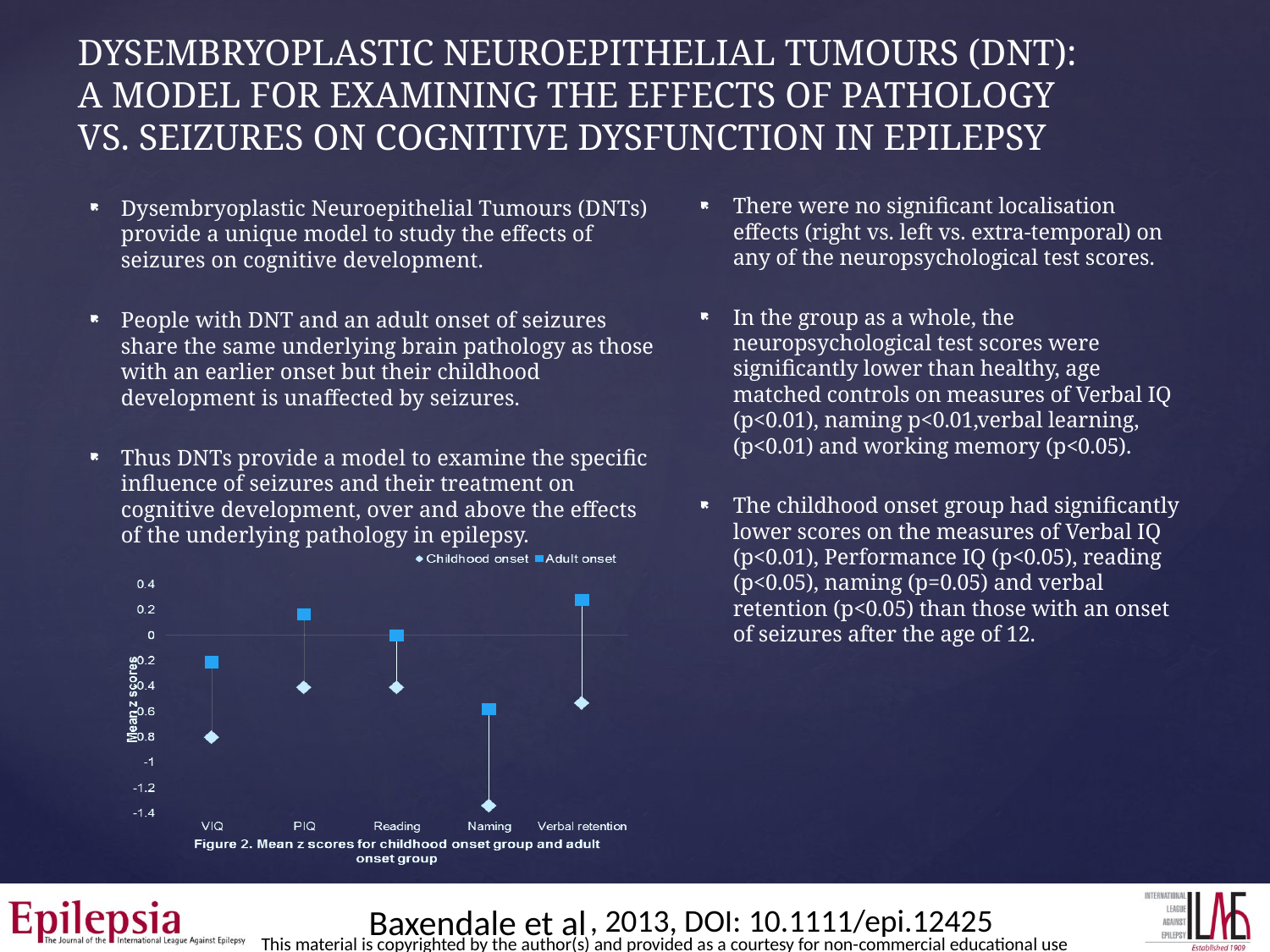

# dysembryoplastic neuroepithelial tumours (DNT): A model for examining the effects of pathology vs. seizures on cognitive dysfunction in Epilepsy
Dysembryoplastic Neuroepithelial Tumours (DNTs) provide a unique model to study the effects of seizures on cognitive development.
People with DNT and an adult onset of seizures share the same underlying brain pathology as those with an earlier onset but their childhood development is unaffected by seizures.
Thus DNTs provide a model to examine the specific influence of seizures and their treatment on cognitive development, over and above the effects of the underlying pathology in epilepsy.
There were no significant localisation effects (right vs. left vs. extra-temporal) on any of the neuropsychological test scores.
In the group as a whole, the neuropsychological test scores were significantly lower than healthy, age matched controls on measures of Verbal IQ (p<0.01), naming p<0.01,verbal learning, (p<0.01) and working memory (p<0.05).
The childhood onset group had significantly lower scores on the measures of Verbal IQ (p<0.01), Performance IQ (p<0.05), reading (p<0.05), naming (p=0.05) and verbal retention (p<0.05) than those with an onset of seizures after the age of 12.
Baxendale et al
, 2013, DOI: 10.1111/epi.12425
This material is copyrighted by the author(s) and provided as a courtesy for non-commercial educational use
